# Epidemic tracking and forecasting: Lessons learned from a tumultuous year

**DOI:** 10.1073/pnas.2111456118

**Published:** 2021-12-13

**Authors:** Roni Rosenfeld, Ryan J. Tibshirani

**Affiliations:** ^a^Machine Learning Department, Carnegie Mellon University, Pittsburgh, PA 15213;; ^b^Department of Statistics and Data Science, Carnegie Mellon University, Pittsburgh, PA 15213

Epidemic forecasting has garnered increasing interest in the last decade, nurtured and scaffolded by various forecasting challenges organized by groups within the US federal government, including the Centers for Disease Control and Prevention (CDC) (1–3), Office of Science and Technology Policy (OSTP) (4), and Defense Advanced Research Projects Agency (DARPA) (5), and elsewhere (6, 7). In 2017, after several years of experimentation with flu forecasting in academic groups, the CDC decided to incorporate influenza forecasting into its normal operations, including weekly public communications (8) and briefing to higher-ups. To provide more reliable infrastructure and support for its forecasting needs, the CDC in 2019 designated two national Centers of Excellence for Influenza Forecasting, one at the University of Massachusetts at Amherst (https://reichlab.io/people) and one at Carnegie Mellon University (https://delphi.cmu.edu/about/center-of-excellence/).

Not unrelatedly, the last decade has also seen a rise in the importance of digital surveillance streams in public health, with improving epidemic tracking and forecasting models being a key application of these data. Digital streams, such as search and social media trends, have constituted a large part of the focus (9–14); however, even more broadly, data from auxiliary streams that operate outside of traditional public health reporting, such as online surveys, medical devices, or electronic medical records (EMRs), have received considerable attention as well (15–25).

The Carnegie Mellon Delphi group, which the two of us colead, has worked in both of these emerging disciplines—epidemic forecasting and building relevant auxiliary signals to aid such forecasting models—since 2012. In 2020, as the pandemic broke out, we struggled like many other groups to find ways to contribute to the national efforts to respond to the pandemic. We ended up shifting our focus to nearly entirely on the data end of the spectrum, pursuing several different directions in order to build and make available to the public a variety of new indicators that reflect real-time COVID-19 activity in the United States. Three papers in this collection describe this work from three different perspectives (26–28). A fourth describes international work by some of our collaborators that parallels our group’s work on online surveys in the United States (29).

## 1. Papers in This Collection

Here is a very brief summary of the papers in this collection.

1) Reinhart et al. (26) describe our group’s (ongoing) effort in building and maintaining COVIDcast: an open repository of real-time, geographically detailed COVID-19 indicators in the United States. These indicators (a term we use interchangeably with signals) are derived from a diverse set of data sources: medical testing devices, medical insurance claims, internet search trends, app-based mobility data, and online surveys among others. Many indicators are demonstrated to have meaningful statistical relationships with what have become the pandemic’s “topline” numbers (reported cases, hospitalizations, deaths), whereas others uniquely reflect certain activity (not available in other publicly available data sources) that may drive or affect the spread of COVID-19. The paper demonstrates through a number of examples that the indicators in the COVIDcast repository can improve on the timeliness, robustness, and scope of traditional public health reporting streams.
2) McDonald et al. (27) provide a detailed analysis of whether a core set of the indicators in the COVIDcast repository can be used to improve the accuracy of COVID-19 short-term forecasting and hot spot detection models. This speaks to the quantitative utility of the indicators in a way that is directly tied to the benefits observed in relevant downstream modeling tasks. The paper finds that time series models [that are already competitive with top forecasters from the COVID-19 Forecast Hub (30)] improve in predictive accuracy when they are supplemented with any of the five indicators under consideration, based on COVID-related medical insurance claims, self-reported symptoms from surveys (in fact, from COVID-19 Trends and Impact Survey [CTIS], described next), and COVID-related Google searches.
3) Salomon et al. (28) focus on the US CTIS, an (ongoing) online survey operated by our group in partnership with Facebook. This is a very rich source of data about the pandemic and its effect on people, only partially reflected by the indicators (derived from the survey) in the COVIDcast repository; the full dataset of individual, anonymized survey responses is available to researchers under a data use agreement. The paper presents descriptive analyses that reflect the unique value of CTIS as an important supplement to public health reporting, in particular as an instrument to measure key information about behaviors, attitudes, economic impacts, and other topics not covered in traditional public health streams.
4) Astley et al. (29) focuses on the international version of CTIS, which is an (ongoing) online survey operated by the University of Maryland, again in partnership with Facebook. This international survey covers over 100 countries and territories, and is run in coordination with the US one, so that the two bear similar structures and undergo similar updates; the full data set of individual, anonymized international survey responses is again available to individual researchers under a data use agreement. The paper presents analyses that reflect some basic and important characteristics of the international survey, reflecting its value abroad, where public health reporting efforts may be more limited than those in the US.

## 2. Lessons Learned

We now take the opportunity to reflect on some “lessons learned” from our work over the past year and a half. Some of the observations below are described in more depth in the papers in the collection, and others extend beyond the papers in the collection (but we give references to relevant articles with more details in the discussion below).

### Deceptively Simple Data Labels Often Belie the Data’s True Meaning and Complexity

Labels such as “COVID-19 cases” or “COVID-19 hospitalizations” hide an enormous amount of complexity and potential ambiguity, especially when applied to data at fine geographic and temporal resolutions. We elaborate on this and several other examples in what follows.

• Cases may be laboratory confirmed only or also suspected (with the definition of “suspected” varying across jurisdictions and time); they may be listed by date reported on the jurisdiction’s website, by date reported to the public health authority, by date tested, by specimen collection date, or occasionally, by symptom onset date (most informative but often unavailable or inapplicable). A casual review of many websites of local and state health departments suggests there is great heterogeneity in what is being reported (31).
• The term “hospitalizations” is used ambiguously, sometimes referring to incidence (hospital admissions) and sometimes to prevalence (hospital bed occupancy). These two quantities cannot easily be mapped to one another because COVID-19 hospital discharges are rarely if ever reported. Furthermore, people admitted without a COVID-19 diagnosis may acquire the infection and/or the diagnosis at any time during their hospital stay.
• Hospitalizations may be reported by the location (typically county) of the patient’s residence but are more often reported by the county of the reporting hospital. This is an important distinction, as many rural COVID-19 patients in need of advanced care travel to the nearest secondary or tertiary hospital, often at a nearby urban county. For example, the hospitals in Pittsburgh, located in Allegheny County, Pennsylvania (population 1.2 million), treat patients from throughout a 13-county region in southwestern Pennsylvania (population over 4 million). For tracking and forecasting hospitalization burden, then the geographic unit of hospital referral regions (32) may be most appropriate. Alas, these units do not conform to county boundaries, which complicates the projection of cases to hospitalizations.
• Deaths are usually reported by county where they occurred, which for hospitalized patients, may differ from their county of residence (33).
• Hospitalization or deaths with COVID-19 are significantly different from hospitalization or deaths due to COVID-19 (as captured by, e.g., a COVID-19–related chief complaint or primary ICD-10 code). The proportion of the two varies significantly by age groups and across time (34).
• Test positivity rates are most often reported by lumping together all tests performed regardless of the reason for performing them. Tests taken following positive diagnosis, due to symptoms, or due to being a contact of a confirmed case are all likely to have a much higher positivity rate than those of the general population. Screening tests are most likely to reflect the true prevalence in the screened population. Sadly, very few jurisdictions report or even retain the breakdown of the test results by reason for testing, thereby losing forever valuable information.
• Medical insurance claims offer rich, detailed information about COVID-19 and other health conditions but are not without their weaknesses. Claims are often not filed until weeks and months after the medical encounter. As such, signals derived from claims are usually subject to regular and considerable revisions up to 60 days after a given date of service because signals must be updated each time new claims for that day of service are received (specific statistics on how this affects signal values are given below). This tends to make projections challenging, especially at finer geographic units such as counties, since there tends to be a high degree of heterogeneity across locations.
• Medical claims contain information about laboratory tests taken but not their results. More generally, for understandable reasons that have to do with the Health Insurance Portability and Accountability Act (HIPAA), medical claims contain only information necessary for adjudicating and auditing claims.

Data definitions must be disambiguated, clarified, and made consistent to the greatest extent possible, and remaining inconsistencies must be documented and saliently communicated.

### Understanding the Data Generation Process Is Critical for Downstream Applications

Both traditional public health surveillance data streams and newer digital surveillance streams are the result of often complex processes, some having to do with the underlying health status or activities being monitored and others with the reporting process itself. Understanding the entire “data generation process” for each data source can be challenging, but it is absolutely essential for proper modeling and effective use of the data. Some examples are as follows.

• In medical claims, relevant diagnoses and comorbidities may not be reflected if they are not directly relevant to the charges incurred. On the other hand, because medical claim coding determines reimbursement levels, some codes may be overrepresented relative to their medical significance.
• Some populations and some health care settings are not reflected in the commercial claims stream. These include the health care systems of the Department of Defense, Indian Health Services, Veterans Affairs, prison systems, and other systems that do not reimburse by procedure or service, as well as Medicare fee for service and Medicaid. This can cause significant bias in the signals relative to the prevalence in the general population.
• Public health reporting data are often subject to backlogs and reporting delays, and estimates for any particular date can be revised over time as errors are found or additional data become available. During the pandemic, audits, corrections, and the clearing of backlogs have frequently resulted in huge artificial spikes and drops (35). Data aggregators like Johns Hopkins Center for Systems Science and Engineering (36) have worked tirelessly to correct such anomalies after first publication (they attempt to back distribute a spike or dip by working with a local authority to figure out how this should best be done).
• Data revisioning (also known as “backfill”) is pervasive not only in traditional public health reporting but also in many (although not all) digital surveillance sources. As already alluded to above, signals based on medical claims typically undergo regular revisions because many claims (on which these signals are based) get submitted and processed late; for many COVID-19–related claims-based signals, the median relative error between initial reports and final values is over 10%, and only after 30 days or so do estimates typically match finalized values within 5% (26). However, the systematic nature of these revisions suggests that, with suitable historical data, statistical models could be fit to estimate the final values from preliminary reports. By comparison, revisions to public health reports during the pandemic (the spikes and dips just described) have been much less systematic and much less predictable.
• Traditionally, public health agencies do not publish provisional data until they meet a level of stability. For example, data from the National Center for Health Statistics (NCHS) on the percentage of deaths due to pneumonia, influenza, and COVID-19 are not released until at least 20% of the expected deaths in a jurisdiction have been reported (37), a process that may take several weeks. However, for modeling and forecasting purposes, even highly provisional data can be very informative, as long as sufficient historical provisional data are collected so that the statistical relationships between provisional values and finalized values can be modeled.
• Calendar effects permeate not only the reporting process but also health-seeking behavior and the epidemic process itself, with the effects on these three processes not easy to disentangle. Major holidays and other national or regional events are associated with significant travel, social mixing, and other distinct behaviors affecting disease transmission. However, holidays and weekends also affect health-seeking behavior via reduced nonemergency health care capacity (doctors’ offices and laboratories being partially or fully closed). Perhaps the strongest calendar effects are on reporting, including claim filing and hospital reporting. Using one- or two-week trailing averages eliminates weekend effects but at a cost of reduced temporal resolution, and it leaves unsolved the effects of holidays. A better approach might be to explicitly model and correct for calendar effects.

### Mandated Reporting in a Time of Emergency Can Be Burdensome and Inflexible

COVID-19 reporting by hospitals, as mandated by the Department of Health and Human Services (HHS) during the pandemic, consisted of many dozens of data elements and imposed a significant burden on the nation’s 6,000 or so hospitals at a time when they were already stretched to their limits. It also took a huge effort to formulate, communicate, disambiguate, and monitor for quality assurance and uniformity of interpretation. In light of this, it is not surprising that it took a long time and pressure for most hospitals to comply (near-universal compliance was not achieved until December 2020). When changes needed to be made to the collected statistics, an arduous and time-consuming process of approvals, reformulation, recommunication, reimplementation, and reassessment had to be followed.

While some aspects of mandated reporting are likely to remain irreplaceable, effective alternative surveillance sources can be of great use; they can improve on the timeliness, scope, robustness, and utility of mandated reporting data while being less burdensome to collect. This is a common theme that runs through all four papers in the collection, but it is perhaps most directly addressed in Reinhart et al. (26) (which focuses on the ecosystem of signals broadly). That said, we have far from saturated the utility of auxiliary surveillance. Much more needs to be developed in this area in order to usher epidemic tracking into its next phase of reliability, accuracy, and transparency. To us, EMRs hold a great promise for surveillance streams, and we elaborate on this in the next section.

### Human Behavior and Its Impact on the Progression of Epidemics Is Hard to Measure and Hard to Model

In the nearly 10 years of government-organized epidemic forecasting exercises in the United States, efforts were focused on modeling the natural history and likely evolution of the pathogen, with adaptation of human behavior playing a secondary role (if any role at all). The pandemic demonstrated that our forecasting models must pay closer attention to reactive human behavior, even more so if we are to consider interventions. Unfortunately, many highly relevant aspects of human behavior, like compliance with policies and recommendations, are not measured by publicly available data streams [with perhaps surveys and mobility reports providing our best glimpse into these hard-to-observe aspects of behavior (38)]. Furthermore, even if we had these data in hand, incorporating their effects will require significant and new cognitive and behavioral modeling, with uncertain success. The tragic breakdown and fragmentation of trust in governments, public health officials, and health care professionals are perhaps the hardest factors to measure and model, yet they played an undeniable role in the progression of the current pandemic in the United States and other countries.

## 3. The Road Ahead

The focus of the Delphi group during the initial critical period of the pandemic (February 2020 to March 2021) was on short-term goals: trying to provide signals, analysis, and decision support to federal, state, and local public health officials, as well as to fellow researchers, data journalists, and the general public. In spring of 2021, equipped with the hard lessons learned during this tumultuous year, we turned our attention back to the original vision of the Delphi group. We asked ourselves the following question: Given where we are and what we know now, what is needed to be able to take a major step forward in epidemic tracking and forecasting?

In this section, we list some ideas, which we hope will elicit further public discussion and most importantly, experimentation. In this regard, the creation of the Center for Forecasting and Analytics at the CDC (39) is a welcome and much anticipated development. Because our expertise lies in modeling and forecasting, not in public health surveillance, our perspective and recommendations in what follows are necessarily limited to those aspects of surveillance that are needed to realize our vision.

### EMR as a Key Missing Component for Epidemic Tracking and Forecasting

The success of nowcasting, analytics, and forecasting depends crucially on the availability of rich, real-time data sources. In light of the limitations of mandated reporting discussed above, we must consider the complementary value of other data sources. Chief among these are EMRs as are being created and used daily by inpatient and outpatient health care providers, medical laboratories, and pharmacies. The advantages of these data resources are that they are rich, real time, and already being generated (found “in the wild”). The challenges are that they are highly fragmented in the United States, with its ∼6,000 hospitals and ∼100,000 outpatient care facilities. One promising avenue for countering this fragmentation is Health Information Exchanges (HIEs), which were set up in the early 2000s with the support of the federal government to coordinate the sharing of health care information among health care providers in a given region and eventually, nationally. The primary goal of the HIEs has been patient continuity of care, but public health surveillance is recognized as an additional worthy goal. In the context of health surveillance, HIEs hold the promise of reducing fragmentation from a hundred thousand partners to only a few hundred.

Other formidable challenges to using EMRs are legal, ethical, commercial, and operational. Who owns the data, who has access to the data, and who has use rights are all complex and often open questions. An overriding concern is, of course, patient privacy. We must find a way to use these highly promising data for the common good without compromising the privacy of individuals. Fortunately, a technological solution appears feasible in the form of federated surveillance. An outline is as follows.

• A common API is developed for querying all participating EMR custodians.
• EMR databases are queried daily with an agreed upon set of queries and return aggregated counts.
• These counts are further aggregated across multiple providers and localities and then, fused with all other available data sources to provide alerts, nowcasts, and forecasts.
• These model outputs are then shared back to the contributing EMR custodians, as well as to the CDC and other federal and state agencies.
• Done in this way, no personal health information ever leaves the custodians’ premises, while aggregated statistics can be combined to increase statistical power, thereby shortening alert latency and improving detection and prediction capabilities.

A successful example of federated querying (albeit designed for research rather than real-time tracking and forecasting) has been recently demonstrated in the United Kingdom (40).

One advantage of this approach to health surveillance is that when a new emerging health crisis is identified or when specific syndromic signatures are discovered (e.g., ageusia and anosmia for COVID-19), a new query can be developed, approved, and deployed literally overnight, allowing us to “shine a light” on it on very short notice. This can be contrasted with traditional, legally mandated public health reporting, which could take weeks and months to develop, approve, negotiate, disseminate, implement, monitor, and assure quality of, as has happened during the current pandemic. In the slightly longer term, demonstrating the effectiveness and superiority of federated surveillance could obviate the need for crisis time mandated reporting, alleviating the reporting burden on hospitals and other health care providers during these difficult times.

In an interpandemic period, an important use case for federated surveillance is detection of trends and anomalies. A set of queries can be designed to continuously test for unusual recent spikes or trends in any number of diagnoses, symptoms, laboratory tests, or prescriptions. The aggregation of evidence across many systems, localities, and data streams will make detection both more sensitive and more robust. Such a system would likely have detected the opioid epidemic years before it was actually noticed.

One open technical challenge with the federated surveillance approach is semantic heterogeneity; the use of emerging Health Information Technology standards like HL7’s FHIR (41) can enable a unified view of EMR data elements, but different health care systems often have different operational definitions for supposedly universal concepts like “high blood pressure” or “low blood oxygenation.” Combining counts of such events across different health care systems may be a bit like adding apples to oranges. It will take some work to harmonize semantics across so many diverse data custodians, but this is both doable and well worth doing. Note that this problem is less severe for the many surveillance and anomaly detection tasks where the focus is on changes in a signal (in a given location) over time, rather than on its absolute meaning.

### Different Phases of Epidemic Surveillance Call for Different Analytic Tools

It is important to discuss analytic needs separately for each of three different phases of epidemic surveillance and tracking since each poses different technical challenges and requires different analytical tools.

In the interpandemic phase, the main activity is threat scanning, namely monitoring data streams and events throughout the world for disconcerting developments. Relevant statistical tools include anomaly detection and scan statistics to help decide when an epidemiological investigation is warranted. While it may be possible to rank the risks of different outbreak triggering events (like species jumping or point mutations) in different locations, which could in turn be used to inform surveillance resource allocation, conventional forecasting has a limited role to play in this phase, as such events have large inherent uncertainty.

In the containment phase, a discovered threat must be intensely monitored, continuously assessed, and ultimately contained. The analytical, data-driven tools required in this phase include real-time estimation of critical epidemiological parameters such as R0, infection fatality rates, the incubation period, the serial interval, and so on. These real-time estimates are necessarily based on provisional data, highlighting the value of modeling the data generation process discussed above. In this phase, forecasting still has a limited role since the outbreak is still local, its fundamental dynamics are unclear, and point events can have large consequences down the road.

If containment fails, during the mitigation phase the goals of analytics expand significantly to include informing mitigation policies and planning. Real-time tracking (nowcasting) and short-term forecasting (a few weeks ahead) can play critical roles in these activities and indeed, have been the focus of our group’s work since its inception. While there is still important work to be done and advances to be made in this area, we believe that these advances are likely to be incremental until we see major progress in either 1) supporting data streams (e.g., better standardization and cleaning of public health reporting data, identification of leading indicators from, say, EMR) or 2) our collective scientific understanding of the real-world geotemporal dynamics of epidemics (discussed next).

### Useful, Reliable Longer-Term Forecasting Remains an Aspiration

Influenza forecasting exercises in the last several years demonstrated that it is often possible to usefully quantify uncertainty over the remainder of an ongoing flu season (3). However, this success was based mostly on observing the behavior of seasonable epidemics over several decades. To reliably forecast the progression of pandemics, where relevant historical data are almost nonexistent, we must have a detailed quantitative understanding of how different, diverse factors affect disease transmissibility. Such an understanding is currently grossly lacking, as evidenced by our collective failure to predict (42) [or even understand post hoc (43)] the high-level temporal and geographic contours of the main pandemic waves in the United States. Yet, this very pandemic, the most instrumented in human history, is also a rare opportunity to attempt this vital scientific and technological goal.
